# *catena*-Poly[[[aqua­tripyridine­cobalt(II)]-μ-5-amino-2,4,6-triiodoisophthalato-κ^2^ *O* ^1^:*O* ^3^] pyridine solvate]

**DOI:** 10.1107/S1600536808032017

**Published:** 2008-10-15

**Authors:** Yu Zhang, Jianying Zhao, Guodong Tang, Zhengjing Jiang

**Affiliations:** a Department of Chemistry, Huaiyin Teachers College, Huai’an 223300, Jiangsu, People’s Republic of China

## Abstract

The reaction of cobalt(II) nitrate with 5-amino-2,4,6-tri­iodo­isophthalic acid (ATPA) in pyridine solution leads to the formation of the title compound, {[Co(C_8_H_2_I_3_NO_4_)(C_5_H_5_N)_3_(H_2_O)]·C_5_H_5_N}_*n*_. The Co^2+^ ion is six-coordinated by three N atoms, one water O atom and two O atoms from two ATPA ligands to form a distorted octa­hedral geometry. The two carboxyl­ate groups of ATPA act as bridging ligands connecting the Co^II^ metal centers to form one-dimensional zigzag chains along the *c* axis, with Co—O distances in the range 2.104 (4)–2.135 (4) Å. The average Co—N distance is 2.171 Å. A classical O—H⋯N hydrogen bond is formed by the coordinating water mol­ecule and the pyridine solvent mol­ecule. The structure was refined from a racemically twinned crystal with a twin ratio of approximately 8:1.

## Related literature

For the structure of a monohydrate of ATPA, see: Beck & Sheldrick (2008 ▶). For the Co coordination polymer of 1,3,5-benzene­tricarboxyl­ate, see: Livage *et al.* (2001 ▶). For the structure of diaqua­diformatodipyridine Co^II^, see: Zhu *et al.* (2004 ▶). For a reduction of the organic iodine contrast agents in wastewater load, see: Ziegler *et al.* (1997 ▶).
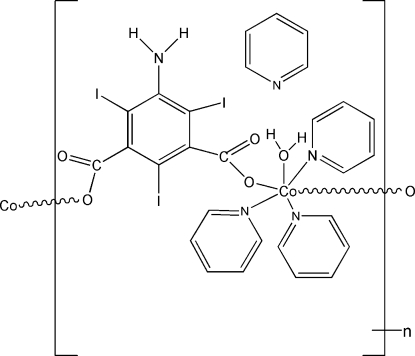


## Experimental

### 

#### Crystal data

[Co(C_8_H_2_I_3_NO_4_)(C_5_H_5_N)_3_(H_2_O)]·C_5_H_5_N
*M* *_r_* = 950.15
Orthorhombic, 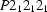

*a* = 9.7759 (2) Å
*b* = 16.9083 (4) Å
*c* = 19.3380 (4) Å
*V* = 3196.45 (12) Å^3^
*Z* = 4
Mo *K*α radiation
μ = 3.48 mm^−1^
*T* = 296 (2) K
0.30 × 0.25 × 0.08 mm

#### Data collection

Bruker APEXII CCD diffractometer
Absorption correction: multi-scan (*SADABS*; Bruker, 2000 ▶) *T*_min_ = 0.38, *T*_max_ = 0.75
16692 measured reflections
6038 independent reflections
4577 reflections with *I* > 2σ(*I*)
*R*_int_ = 0.027

#### Refinement

*R*[*F* ^2^ > 2σ(*F* ^2^)] = 0.041
*wR*(*F* ^2^) = 0.065
*S* = 1.04
6038 reflections
379 parameters
3 restraints
H-atom parameters constrained
Δρ_max_ = 0.68 e Å^−3^
Δρ_min_ = −0.67 e Å^−3^
Absolute structure: Flack (1983 ▶), with 2515 Friedel pairs
Flack parameter: 0.13 (2)

### 

Data collection: *APEX2* (Bruker, 2004 ▶); cell refinement: *SAINT* (Bruker, 2004 ▶); data reduction: *SAINT*; program(s) used to solve structure: *SHELXS97* (Sheldrick, 2008 ▶); program(s) used to refine structure: *SHELXL97* (Sheldrick, 2008 ▶); molecular graphics: *ORTEP-3 for Windows* (Farrugia, 1997 ▶); software used to prepare material for publication: *SHELXL97* and *PLATON* (Spek, 2003 ▶).

## Supplementary Material

Crystal structure: contains datablocks I, global. DOI: 10.1107/S1600536808032017/si2113sup1.cif

Structure factors: contains datablocks I. DOI: 10.1107/S1600536808032017/si2113Isup2.hkl

Additional supplementary materials:  crystallographic information; 3D view; checkCIF report

## Figures and Tables

**Table:** 

Co1—O1^i^	2.104 (4)
Co1—O5	2.106 (3)
Co1—O3	2.135 (4)
Co1—N2	2.161 (5)
Co1—N3	2.173 (5)
Co1—N4	2.180 (5)

**Table:** 

O1^i^—Co1—O3	170.52 (16)
O1^i^—Co1—N3	102.93 (17)
O5—Co1—N3	172.68 (17)
N2—Co1—N4	178.48 (19)

Symmetry code: (i) 
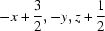
.

**Table 2:** Hydrogen-bond geometry (Å, °)

*D*—H⋯*A*	*D*—H	H⋯*A*	*D*⋯*A*	*D*—H⋯*A*
O5—H5*A*⋯N5^ii^	0.85	1.94	2.748 (7)	159

Symmetry code: (ii) 
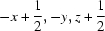
.

**Fig. 1.**
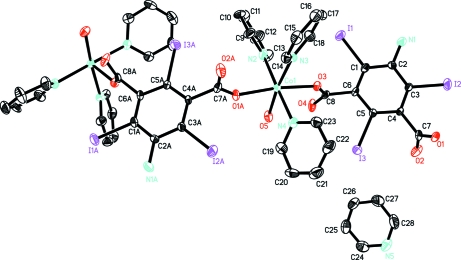
The molecular structure of (I), with atom labels and 30% probability displacement ellipsoids for non-H atoms. Atoms labelled with an A belong to the symmetry-related ligand ATPA with symmetry code [A = -x + 3/2, -y, z + 1/2)].

## supplementary crystallographic information

### Comment

The crystal structure of ATPA (Beck & Sheldrick, 2008) is the precursor of the synthesis of a wide range of contrast agents with different amide-bound aliphatic side chains, which modulate their physical and physiological properties (Ziegler *et al.* 1997). However, to the best of our knowledge, there is no information about the structural characterization of its transition metal complexes.

The molecular structure of the title complex comprises of polymeric chains which extend along the *c*-axis. In the chain, each Co atom shows a distorted octahedron environment with a [3N+3O] coordination: three nitrogen atoms originate from pyridines, one oxygen from a water molecule and two oxygen atoms from two ATPA ligands. The two CO_2_^-^ groups of the ATPA ligand coordinate to Co^2+^, bridging the Co metal centers. The bond lengths of the distorted octahedron are presented in Table 1. The average Co—N bond distance of the three pyridine ligands is 2.171 Å. The Co—O bond lengths in the title complex are slightly longer than those in the reported coordination polymers of cobalt and 1,3,5-benzenetricarboxylate (2.055 (2) Å) (Livage *et al.*, 2001). The bond angles shown in Table 1 demonstrate the distorted octahedron in the Co coordination center. Compared with the data of the free ligand ATPA (Beck & Sheldrick, 2008), the C—O bond lengths are lengthened, the C—I and C—N bond distances are almost unchanged and the O—C—O bond angles are slightly expanded when the carboxylate groups are coordinated to central cations. The Co—N(py) and Co—O(H_2_O) distances are in good agreement with those in diaqua-diformato-dipyridine-cobalt(II) (Zhu *et al.*, 2004), where they are equal to 2.159 (4) Å and 2.143 (3) Å, respectively. A classic O—H···N hydrogen bond is formed by the coordinating water and the uncoordinated pyridine molecule (Table 2).

### Experimental

0.29 g (1 mmol) Co(NO_3_)_2_.6H_2_O was dissolved in 10 ml ethanol, 0.54 g (1 mmol) 5-amino-2, 4, 6-triiodoisophthalic acid was dissolved in 10 ml pyridine. To mix two solutions gave a pale purple solution which was stirred at room temperature for 2 h, then filtered. After several days well formed light purple single crystals were obtained.

### Refinement

H atoms were positioned geometrically and refined using a riding model with C—H distances = 0.93 Å, N—H distances = 0.86 Å, and O—H distances = 0.85 Å with *U*_iso_(H) = 1.2 times *U*_eq_(C, N, O).

### Crystal data

**Table:**

[Co(C_8_H2I_3_NO_4_)(C_5_H_5_N)_3_(H_2_O)]·C_5_H_5_N	*F*(000) = 1812
*M**_r_* = 950.15	*D*_x_ = 1.974 Mg m^−^^3^
Orthorhombic, *P*2_1_2_1_2_1_	Mo *K*α radiation, λ = 0.71073 Å
Hall symbol: P 2ac 2ab	Cell parameters from 7120 reflections
*a* = 9.7759 (2) Å	θ = 4.7–43.0°
*b* = 16.9083 (4) Å	µ = 3.48 mm^−^^1^
*c* = 19.3380 (4) Å	*T* = 296 K
*V* = 3196.45 (12) Å^3^	Sheet, light purple
*Z* = 4	0.30 × 0.25 × 0.08 mm

### Data collection

**Table:**

Bruker APEXII CCD diffractometer	6038 independent reflections
Radiation source: fine-focus sealed tube	4577 reflections with *I* > 2σ(*I*)
graphite	*R*_int_ = 0.027
φ and ω scans	θ_max_ = 26.0°, θ_min_ = 1.6°
Absorption correction: multi-scan (SADABS; Bruker, 2000)	*h* = −12→9
*T*_min_ = 0.38, *T*_max_ = 0.75	*k* = −13→20
16692 measured reflections	*l* = −15→23

### Refinement

**Table:**

Refinement on *F*^2^	Secondary atom site location: difference Fourier map
Least-squares matrix: full	Hydrogen site location: inferred from neighbouring sites
*R*[*F*^2^ > 2σ(*F*^2^)] = 0.041	H-atom parameters constrained
*wR*(*F*^2^) = 0.065	*w* = 1/[σ^2^(*F*_o_^2^) + (0.0243*P*)^2^] where *P* = (*F*_o_^2^ + 2*F*_c_^2^)/3
*S* = 1.04	(Δ/σ)_max_ = 0.001
6038 reflections	Δρ_max_ = 0.68 e Å^−^^3^
379 parameters	Δρ_min_ = −0.67 e Å^−^^3^
3 restraints	Absolute structure: Flack (1983), with 2515 Friedel pairs
Primary atom site location: structure-invariant direct methods	Flack parameter: 0.13 (2)

### Special details

**Table:**

Geometry. All e.s.d.'s (except the e.s.d. in the dihedral angle between two l.s. planes) are estimated using the full covariance matrix. The cell e.s.d.'s are taken into account individually in the estimation of e.s.d.'s in distances, angles and torsion angles; correlations between e.s.d.'s in cell parameters are only used when they are defined by crystal symmetry. An approximate (isotropic) treatment of cell e.s.d.'s is used for estimating e.s.d.'s involving l.s. planes.
Refinement. Refinement of *F*^2^ against ALL reflections. The weighted *R*-factor *wR* and goodness of fit *S* are based on *F*^2^, conventional *R*-factors *R* are based on *F*, with *F* set to zero for negative *F*^2^. The threshold expression of *F*^2^ > σ(*F*^2^) is used only for calculating *R*-factors(gt) *etc*. and is not relevant to the choice of reflections for refinement. *R*-factors based on *F*^2^ are statistically about twice as large as those based on *F*, and *R*- factors based on ALL data will be even larger.

### Fractional atomic coordinates and isotropic or equivalent isotropic displacement parameters (Å^2^)

**Table:**

	*x*	*y*	*z*	*U*_iso_*/*U*_eq_	
C1	0.8251 (6)	0.2139 (3)	0.8550 (3)	0.0319 (15)	
C2	0.8423 (6)	0.2494 (3)	0.7900 (3)	0.0344 (16)	
C3	0.7752 (6)	0.2152 (3)	0.7335 (3)	0.0299 (15)	
C4	0.6949 (6)	0.1483 (3)	0.7390 (3)	0.0229 (14)	
C5	0.6819 (6)	0.1159 (3)	0.8047 (3)	0.0286 (15)	
C6	0.7445 (6)	0.1478 (3)	0.8629 (3)	0.0243 (14)	
C7	0.6216 (7)	0.1144 (3)	0.6765 (3)	0.0276 (15)	
C8	0.7222 (7)	0.1122 (3)	0.9341 (3)	0.0305 (15)	
C9	0.9902 (7)	0.0943 (4)	1.1534 (4)	0.052 (2)	
H9	1.0468	0.0501	1.1540	0.063*	
C10	1.0171 (8)	0.1565 (5)	1.1968 (4)	0.067 (2)	
H10	1.0909	0.1528	1.2270	0.080*	
C11	0.9406 (10)	0.2230 (5)	1.1973 (4)	0.078 (3)	
H11	0.9607	0.2651	1.2266	0.093*	
C12	0.8332 (9)	0.2254 (5)	1.1531 (4)	0.078 (3)	
H12	0.7768	0.2697	1.1520	0.093*	
C13	0.8073 (7)	0.1626 (4)	1.1100 (4)	0.060 (2)	
H13	0.7342	0.1661	1.0793	0.072*	
C14	1.1101 (6)	−0.0851 (4)	1.0269 (3)	0.0397 (18)	
H14	1.0619	−0.1279	1.0447	0.048*	
C15	1.2437 (7)	−0.0963 (4)	1.0090 (3)	0.0488 (19)	
H15	1.2859	−0.1451	1.0151	0.059*	
C16	1.3142 (7)	−0.0329 (5)	0.9817 (3)	0.053 (2)	
H16	1.4044	−0.0391	0.9674	0.064*	
C17	1.2528 (6)	0.0377 (4)	0.9759 (3)	0.0471 (19)	
H17	1.3000	0.0815	0.9592	0.057*	
C18	1.1168 (7)	0.0434 (4)	0.9955 (3)	0.0448 (18)	
H18	1.0733	0.0921	0.9911	0.054*	
C19	0.6591 (7)	−0.1424 (4)	0.9923 (4)	0.0470 (19)	
H19	0.6114	−0.1305	1.0326	0.056*	
C20	0.6061 (7)	−0.1985 (4)	0.9494 (4)	0.057 (2)	
H20	0.5249	−0.2240	0.9608	0.068*	
C21	0.6717 (9)	−0.2166 (4)	0.8905 (4)	0.069 (2)	
H21	0.6357	−0.2536	0.8599	0.082*	
C22	0.7916 (9)	−0.1797 (4)	0.8766 (4)	0.063 (2)	
H22	0.8416	−0.1925	0.8372	0.076*	
C23	0.8384 (7)	−0.1222 (4)	0.9223 (4)	0.052 (2)	
H23	0.9188	−0.0956	0.9113	0.062*	
C24	−0.0436 (7)	0.0013 (4)	0.7430 (5)	0.062 (2)	
H24	−0.1309	−0.0202	0.7465	0.074*	
C25	0.0132 (9)	0.0325 (5)	0.8004 (4)	0.064 (2)	
H25	−0.0337	0.0321	0.8422	0.076*	
C26	0.1384 (11)	0.0640 (5)	0.7959 (5)	0.096 (3)	
H26	0.1804	0.0855	0.8348	0.115*	
C27	0.2020 (9)	0.0641 (6)	0.7351 (5)	0.101 (3)	
H27	0.2884	0.0867	0.7309	0.121*	
C28	0.1404 (9)	0.0313 (5)	0.6790 (4)	0.082 (3)	
H28	0.1862	0.0311	0.6369	0.098*	
Co1	0.83021 (7)	−0.00406 (5)	1.04654 (4)	0.0312 (2)	
I1	0.92570 (5)	0.26385 (3)	0.94026 (2)	0.05269 (14)	
I2	0.78262 (5)	0.27575 (3)	0.63848 (2)	0.05874 (16)	
I3	0.56454 (5)	0.01146 (3)	0.81795 (2)	0.05039 (14)	
N1	0.9245 (6)	0.3143 (3)	0.7821 (3)	0.0630 (17)	
H1A	0.9357	0.3349	0.7418	0.076*	
H1B	0.9649	0.3345	0.8174	0.076*	
N2	0.8833 (5)	0.0964 (3)	1.1102 (2)	0.0393 (14)	
N3	1.0452 (5)	−0.0175 (3)	1.0205 (2)	0.0348 (13)	
N4	0.7763 (6)	−0.1033 (3)	0.9799 (3)	0.0379 (14)	
N5	0.0172 (7)	−0.0004 (4)	0.6829 (3)	0.0660 (19)	
O1	0.6899 (4)	0.0730 (2)	0.6366 (2)	0.0386 (11)	
O2	0.5004 (5)	0.1311 (3)	0.6709 (2)	0.0555 (14)	
O3	0.8147 (4)	0.0677 (2)	0.95601 (19)	0.0329 (10)	
O4	0.6139 (4)	0.1298 (2)	0.9646 (2)	0.0460 (12)	
O5	0.6191 (3)	0.0161 (2)	1.05937 (18)	0.0423 (11)	
H5B	0.5951	0.0603	1.0423	0.051*	
H5A	0.5963	0.0143	1.1018	0.051*	

### Atomic displacement parameters (Å^2^)

**Table:**

	*U*^11^	*U*^22^	*U*^33^	*U*^12^	*U*^13^	*U*^23^
C1	0.041 (4)	0.029 (4)	0.026 (4)	0.003 (3)	−0.003 (3)	−0.007 (3)
C2	0.043 (4)	0.029 (4)	0.032 (4)	−0.014 (3)	0.003 (3)	−0.004 (3)
C3	0.038 (3)	0.029 (4)	0.023 (3)	−0.005 (3)	−0.004 (3)	0.001 (3)
C4	0.026 (4)	0.022 (3)	0.020 (3)	0.004 (3)	−0.001 (3)	−0.007 (3)
C5	0.032 (3)	0.032 (4)	0.022 (4)	−0.002 (3)	0.003 (3)	−0.003 (3)
C6	0.033 (4)	0.022 (3)	0.017 (3)	0.006 (3)	−0.004 (3)	−0.002 (3)
C7	0.041 (4)	0.020 (4)	0.021 (4)	0.010 (3)	0.005 (3)	0.004 (3)
C8	0.045 (4)	0.027 (4)	0.019 (4)	−0.006 (3)	−0.005 (4)	−0.007 (3)
C9	0.050 (4)	0.053 (5)	0.053 (5)	−0.004 (4)	−0.023 (4)	0.006 (4)
C10	0.078 (6)	0.071 (6)	0.052 (5)	−0.045 (5)	−0.022 (5)	−0.004 (5)
C11	0.122 (8)	0.054 (6)	0.058 (5)	−0.033 (6)	0.009 (6)	−0.022 (5)
C12	0.103 (7)	0.056 (6)	0.075 (6)	0.004 (5)	−0.025 (5)	−0.024 (5)
C13	0.067 (5)	0.054 (5)	0.059 (5)	0.011 (5)	−0.006 (5)	−0.020 (4)
C14	0.042 (4)	0.043 (4)	0.035 (4)	0.004 (4)	0.001 (3)	0.000 (4)
C15	0.046 (5)	0.057 (5)	0.043 (5)	0.020 (4)	0.002 (4)	−0.008 (4)
C16	0.028 (4)	0.088 (6)	0.044 (5)	0.013 (4)	0.003 (3)	−0.009 (4)
C17	0.032 (4)	0.064 (6)	0.045 (4)	−0.008 (4)	−0.001 (3)	0.010 (4)
C18	0.046 (5)	0.041 (4)	0.048 (5)	0.001 (4)	0.003 (4)	0.004 (4)
C19	0.048 (5)	0.037 (4)	0.056 (5)	0.005 (4)	0.003 (4)	0.004 (4)
C20	0.047 (5)	0.043 (5)	0.080 (6)	−0.017 (4)	−0.010 (4)	0.001 (5)
C21	0.088 (7)	0.050 (5)	0.068 (5)	−0.009 (5)	−0.026 (5)	−0.008 (5)
C22	0.085 (6)	0.060 (6)	0.046 (5)	0.000 (5)	−0.004 (5)	−0.028 (4)
C23	0.055 (5)	0.051 (5)	0.049 (5)	−0.011 (4)	0.006 (4)	−0.005 (4)
C24	0.041 (5)	0.061 (5)	0.083 (6)	0.005 (4)	0.005 (5)	−0.002 (6)
C25	0.078 (6)	0.067 (6)	0.046 (5)	0.019 (5)	0.010 (5)	0.001 (5)
C26	0.115 (9)	0.122 (8)	0.050 (6)	−0.044 (7)	−0.034 (6)	0.010 (6)
C27	0.059 (6)	0.167 (10)	0.078 (7)	−0.047 (6)	−0.011 (6)	0.020 (8)
C28	0.063 (6)	0.131 (9)	0.051 (6)	−0.003 (6)	0.014 (5)	0.023 (6)
Co1	0.0333 (4)	0.0350 (5)	0.0254 (5)	−0.0001 (4)	−0.0013 (4)	0.0022 (5)
I1	0.0737 (3)	0.0481 (3)	0.0363 (3)	−0.0171 (3)	−0.0127 (3)	−0.0058 (2)
I2	0.0883 (4)	0.0569 (3)	0.0311 (3)	−0.0191 (3)	0.0001 (3)	0.0134 (3)
I3	0.0633 (3)	0.0495 (3)	0.0384 (3)	−0.0231 (3)	−0.0053 (2)	0.0067 (2)
N1	0.092 (4)	0.062 (4)	0.035 (3)	−0.045 (4)	−0.014 (4)	0.007 (3)
N2	0.048 (4)	0.042 (4)	0.027 (3)	−0.004 (3)	0.000 (3)	−0.001 (3)
N3	0.031 (3)	0.038 (3)	0.035 (3)	0.002 (3)	0.003 (2)	0.006 (3)
N4	0.043 (3)	0.039 (4)	0.031 (3)	0.003 (3)	0.002 (3)	−0.002 (3)
N5	0.063 (4)	0.089 (5)	0.047 (4)	0.014 (4)	−0.012 (4)	−0.011 (4)
O1	0.047 (3)	0.041 (3)	0.028 (2)	−0.001 (2)	0.002 (2)	−0.013 (2)
O2	0.048 (3)	0.075 (4)	0.044 (3)	0.015 (3)	−0.012 (3)	−0.020 (3)
O3	0.037 (3)	0.036 (3)	0.026 (2)	0.001 (2)	−0.005 (2)	0.007 (2)
O4	0.049 (3)	0.064 (3)	0.025 (3)	0.021 (2)	0.010 (2)	0.001 (2)
O5	0.045 (3)	0.048 (3)	0.034 (2)	0.007 (2)	0.006 (2)	0.010 (2)

### Geometric parameters (Å, °)

**Table:**

C1—C6	1.376 (7)	C18—N3	1.336 (7)
C1—C2	1.404 (7)	C18—H18	0.9300
C1—I1	2.097 (6)	C19—N4	1.344 (7)
C2—N1	1.368 (7)	C19—C20	1.363 (9)
C2—C3	1.400 (7)	C19—H19	0.9300
C3—C4	1.381 (7)	C20—C21	1.342 (9)
C3—I2	2.105 (5)	C20—H20	0.9300
C4—C5	1.389 (7)	C21—C22	1.355 (9)
C4—C7	1.517 (8)	C21—H21	0.9300
C5—C6	1.390 (7)	C22—C23	1.391 (8)
C5—I3	2.122 (6)	C22—H22	0.9300
C6—C8	1.519 (4)	C23—N4	1.309 (7)
C7—O2	1.223 (6)	C23—H23	0.9300
C7—O1	1.238 (6)	C24—N5	1.306 (8)
C8—O4	1.248 (6)	C24—C25	1.348 (9)
C8—O3	1.250 (6)	C24—H24	0.9300
C9—N2	1.337 (7)	C25—C26	1.338 (10)
C9—C10	1.372 (9)	C25—H25	0.9300
C9—H9	0.9300	C26—C27	1.331 (11)
C10—C11	1.350 (10)	C26—H26	0.9300
C10—H10	0.9300	C27—C28	1.359 (11)
C11—C12	1.355 (10)	C27—H27	0.9300
C11—H11	0.9300	C28—N5	1.321 (9)
C12—C13	1.373 (9)	C28—H28	0.9300
C12—H12	0.9300	Co1—O1^i^	2.104 (4)
C13—N2	1.345 (7)	Co1—O5	2.106 (3)
C13—H13	0.9300	Co1—O3	2.135 (4)
C14—N3	1.312 (7)	Co1—N2	2.161 (5)
C14—C15	1.365 (8)	Co1—N3	2.173 (5)
C14—H14	0.9300	Co1—N4	2.180 (5)
C15—C16	1.379 (9)	N1—H1A	0.8600
C15—H15	0.9300	N1—H1B	0.8600
C16—C17	1.342 (8)	O1—Co1^ii^	2.104 (4)
C16—H16	0.9300	O5—H5B	0.8500
C17—C18	1.386 (8)	O5—H5A	0.8499
C17—H17	0.9300		
			
C6—C1—C2	121.0 (5)	C19—C20—H20	120.2
C6—C1—I1	120.6 (4)	C20—C21—C22	118.4 (8)
C2—C1—I1	118.4 (4)	C20—C21—H21	120.8
N1—C2—C3	121.3 (5)	C22—C21—H21	120.8
N1—C2—C1	120.9 (5)	C21—C22—C23	118.7 (8)
C3—C2—C1	117.8 (5)	C21—C22—H22	120.6
C4—C3—C2	123.0 (5)	C23—C22—H22	120.6
C4—C3—I2	119.1 (4)	N4—C23—C22	124.0 (7)
C2—C3—I2	117.7 (4)	N4—C23—H23	118.0
C3—C4—C5	116.5 (5)	C22—C23—H23	118.0
C3—C4—C7	121.1 (5)	N5—C24—C25	123.6 (7)
C5—C4—C7	122.5 (5)	N5—C24—H24	118.2
C4—C5—C6	123.2 (5)	C25—C24—H24	118.2
C4—C5—I3	119.2 (4)	C26—C25—C24	118.6 (8)
C6—C5—I3	117.6 (4)	C26—C25—H25	120.7
C1—C6—C5	118.5 (5)	C24—C25—H25	120.7
C1—C6—C8	120.3 (5)	C27—C26—C25	119.0 (9)
C5—C6—C8	121.1 (5)	C27—C26—H26	120.5
O2—C7—O1	126.7 (7)	C25—C26—H26	120.5
O2—C7—C4	116.2 (6)	C26—C27—C28	119.9 (8)
O1—C7—C4	117.2 (5)	C26—C27—H27	120.1
O4—C8—O3	126.7 (5)	C28—C27—H27	120.1
O4—C8—C6	117.0 (6)	N5—C28—C27	121.6 (8)
O3—C8—C6	116.2 (5)	N5—C28—H28	119.2
N2—C9—C10	120.7 (7)	C27—C28—H28	119.2
N2—C9—H9	119.6	O1^i^—Co1—O5	84.29 (15)
C10—C9—H9	119.6	O1^i^—Co1—O3	170.52 (16)
C11—C10—C9	122.5 (8)	O5—Co1—O3	86.29 (15)
C11—C10—H10	118.7	O1^i^—Co1—N2	89.21 (16)
C9—C10—H10	118.7	O5—Co1—N2	92.37 (17)
C10—C11—C12	116.7 (8)	O3—Co1—N2	92.18 (17)
C10—C11—H11	121.7	O1^i^—Co1—N3	102.93 (17)
C12—C11—H11	121.7	O5—Co1—N3	172.68 (17)
C11—C12—C13	120.2 (8)	O3—Co1—N3	86.47 (17)
C11—C12—H12	119.9	N2—Co1—N3	88.95 (19)
C13—C12—H12	119.9	O1^i^—Co1—N4	92.29 (17)
N2—C13—C12	122.7 (7)	O5—Co1—N4	87.53 (18)
N2—C13—H13	118.7	O3—Co1—N4	86.31 (16)
C12—C13—H13	118.7	N2—Co1—N4	178.48 (19)
N3—C14—C15	124.1 (6)	N3—Co1—N4	91.0 (2)
N3—C14—H14	118.0	C2—N1—H1A	120.0
C15—C14—H14	118.0	C2—N1—H1B	120.0
C14—C15—C16	117.8 (7)	H1A—N1—H1B	120.0
C14—C15—H15	121.1	C9—N2—C13	117.2 (6)
C16—C15—H15	121.1	C9—N2—Co1	121.5 (5)
C17—C16—C15	120.0 (6)	C13—N2—Co1	121.3 (5)
C17—C16—H16	120.0	C14—N3—C18	116.9 (5)
C15—C16—H16	120.0	C14—N3—Co1	122.5 (4)
C16—C17—C18	117.9 (7)	C18—N3—Co1	120.7 (4)
C16—C17—H17	121.0	C23—N4—C19	115.3 (6)
C18—C17—H17	121.0	C23—N4—Co1	125.4 (5)
N3—C18—C17	123.2 (6)	C19—N4—Co1	118.7 (5)
N3—C18—H18	118.4	C24—N5—C28	117.2 (7)
C17—C18—H18	118.4	C7—O1—Co1^ii^	141.2 (4)
N4—C19—C20	123.9 (7)	C8—O3—Co1	132.1 (4)
N4—C19—H19	118.0	Co1—O5—H5B	111.5
C20—C19—H19	118.0	Co1—O5—H5A	111.4
C21—C20—C19	119.6 (7)	H5B—O5—H5A	109.4
C21—C20—H20	120.2		

Symmetry codes: (i) −*x*+3/2, −*y*, *z*+1/2; (ii) −*x*+3/2, −*y*, *z*−1/2.

### Hydrogen-bond geometry (Å, °)

**Table:**

*D*—H···*A*	*D*—H	H···*A*	*D*···*A*	*D*—H···*A*
O5—H5A···N5^iii^	0.85	1.94	2.748 (7)	159

Symmetry codes: (iii) −*x*+1/2, −*y*, *z*+1/2.
